# Alternative *REST* Splicing Underappreciated

**DOI:** 10.1523/ENEURO.0034-18.2018

**Published:** 2018-10-01

**Authors:** Guo-Lin Chen, Gregory M. Miller

**Affiliations:** 1 Guangxi Collaborative Innovation Center for Biomedicine, Guangxi Medical University, Nanning, Guangxi 530021, China; 2 Harvard Medical School, New England Primate Research Center, Southborough, MA 01772; 3 Center for Drug Discovery, Department of Pharmaceutical Sciences, School of Pharmacy, Northeastern University, Boston, MA 02115; 4 Department of Chemical Engineering School of Engineering, Northeastern University, Boston, MA 02115

**Keywords:** alternative splicing, epigenome, gene expression, NRSF, REST, RNA process

As a major orchestrator of the cellular epigenome, the repressor element-1 silencing transcription factor (REST) can either repress or activate thousands of genes depending on cellular context, suggesting a highly context-dependent REST function tuned by environmental cues. While *REST* shows cell-type non-selective active transcription (Kojima et al., 2001), an N-terminal REST4 isoform caused by alternative splicing, inclusion of an extra exon (N_3c_) which introduces a pre-mature stop codon, contributes to neurogenesis and tumorigenesis (Palm et al., 1998, 1999; Lee et al., 2000; Raj et al., 2011). Recently, in line with established epigenetic regulation of pre-mRNA splicing (Alló et al., 2010; Luco et al., 2011), we demonstrated that *REST* undergoes extensive, context-dependent alternative splicing which results in the formation of a large number of mRNA variants predictive of multiple protein isoforms (Chen and Miller, 2013). Supported by the fact that immunoblotting/-staining with different anti-REST antibodies yield different results, alternative splicing allows production of various structurally and functionally different REST protein isoforms in response to shifting physiologic requirements, shedding light on environmental regulation of REST function. However, REST isoforms might be differentially assayed or manipulated, leading to data misinterpretation and controversial findings. For example, in contrast to the proposed neurotoxicity of elevated nuclear REST in ischemia (Noh et al., 2012) and Huntington’s disease (Zuccato et al., 2003; Buckley et al., 2010), Lu et al. recently reported decreased nuclear REST in Alzheimer’s disease and neuroprotection of REST in aging brain (Lu et al., 2014). Unfortunately, alternative *REST* splicing was largely neglected by Lu et al. (2014), making it necessary for a reevaluation of their findings.

As shown in Figure 1*A*, human *REST* gene boundary is now doubled by an alternative last exon (E_5_), which is mutually exclusive to E_4_. While numerous novel alternative exons and 5'/3' ends were identified, the three constitutive exons (E_2_, E_3_ and E_4_) comprising the open reading frame (ORF) of *REST* can be skipped partially or completely, alone or in combination, producing at least 45 mRNA variants predictive of multiple protein isoforms (Fig. 1*B*; Chen and Miller, 2013). For example, REST4, which was first described in rat as a group of REST isoforms (Palm et al., 1998), is predicted by multiple mRNA variants (e.g., JX896958, JX896971, and JX896983) with E_3_ followed by variable exons that introduce a pre-mature stop codon. Accordingly, like the case in rat, human REST4 is also a group of isoforms produced by variable splicing predictive of C-terminal truncated proteins that share the same functional domains (RD1 and ZFs 1–5; Fig. 1*B*), and it should no longer be considered as a single mRNA/protein isoform. Meanwhile, REST1, another N-terminal REST isoform, is predicted by multiple mRNA variants lacking E_3_. In addition, for the ubiquitously distributed E_2_-skipped variants (e.g., XM_005265760 and JX896960) missing the conventional start codon, an in-frame AUG in E_3_ may initiate translation of a C-terminal REST^C^ isoform (XP_005265817), which was recently described in *Rest* conditional knock-out (cKO) mice (Nechiporuk et al., 2016), while some partial E_2_-skipped variants (e.g., JX896978 and KC117266) containing the conventional start codon are predictive of proteins missing variable regions of REST. Moreover, it was recently demonstrated that mRNAs with short ORF but previously annotated as noncoding RNAs can actually encode tiny peptides (Magny et al., 2013; Olexiouk et al., 2015; Nelson et al., 2016), such might be the case for numerous REST variants (e.g., JX896962, JX896965, and JX896967). Taken together, REST protein isoforms caused by alternative splicing are much more complex than we expected.

**Figure 1.**
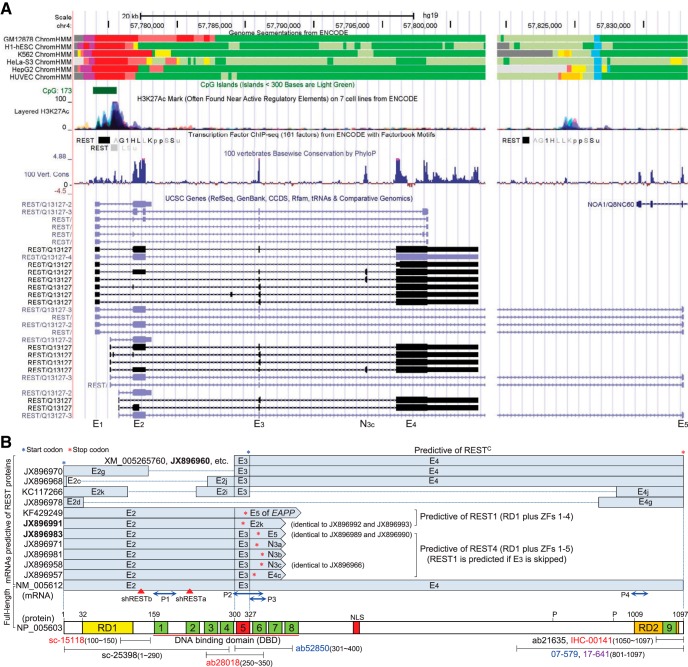
Bioinformatics at human *REST* locus (***A***) and predicted REST protein isoforms derived from alternative splicing (***B***). Related tracks were retrieved from the UCSC Genome Browser (http://genome.ucsc.edu/cgi-bin/hgGateway). *REST* gene boundary is more than doubled by an alternate last exon (E_5_) which partially overlaps in opposite direction with exon 5 of *NOA1*. *REST* promoter harbors a CpG island and exhibits cell-independent active transcription as indicated by the chromatin state segmentation and H3K27Ac tracks. Predicted ORFs of the full-length and alternatively spliced REST mRNAs were briefly shown by indicating the start (blue star) and stop (red star) codons, while major domains (RD1 and RD2, repression domain 1 and 2; NLS, nuclear localization signal; and zinc fingers 1–9) of the full-length REST protein were illustrated in parallel to their coding sequences. Splice variants expressed in multiple tissues or cell lines were bolded. Locations of the mRNA and protein fragments targeted by real-time PCR primer sets (P1–P4), RNAi (shRESTa and shRESTb), and antibodies mentioned in the text were indicated. Note that only the conventional promoter is shown and that the internal region of E_4_ is unconserved as indicated by the “100 vertebrate conservation” track, which supports our finding that partial skipping of E_4_ is common (Chen and Miller, 2013).

Because of the existence of multiple REST mRNA and protein isoforms, it can be inferred that assay of REST expression by different primers (or probes) and antibodies may target different REST isoforms, while manipulation of REST expression by cKO or RNAi may be effective for specific but not all REST variants. In other words, REST isoforms might be differentially assayed or manipulated in different studies, leading to inconsistent results and data misinterpretation. In support of this notion, immunostaining of multiple cell lines with two widely used antibodies, sc-25398 and ab21635 raised against N and C terminus of REST, respectively, produced inconsistent results in terms of REST subcellular distribution and its colocalization with microtubule (Shimojo, 2008; Buckley et al., 2010; Fig. 2), while immunoblotting [i.e., Western blotting (WB)] with the two antibodies yielded different profiles of immunoreactive (IR) bands (Fig. 3), such is the case for some other commercial anti-REST antibodies as described by manufacturer’s manual. Unfortunately, despite the mRNA evidence, not all REST protein isoforms have been experimentally verified and normally they are not observed as expected sizes due to post-translational modifications, making it challenging to determine whether an unknown IR band is non-specific or a REST isoform. For example, REST4 and REST^C^ are predicted as 37 and 86 kDa but observed as 53 and 130 kDa, respectively (Lee et al., 2000; Nechiporuk et al., 2016), while the full-length REST has been reported as variable sizes ranging from 120 to 200 kDa (Liang et al., 2011; Zhang et al., 2011; Nechiporuk et al., 2016). So, even if detectable by WB, specific REST isoforms might be simply considered as non-specific and excluded from being presented in publication, such may explain why REST^C^ was not reported until recently.

**Figure 2.**
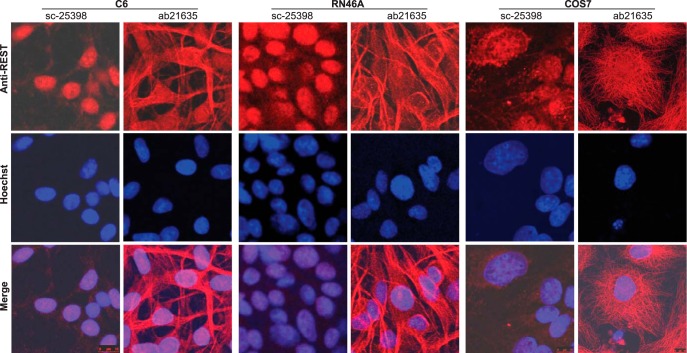
Immunofluorescence analysis of REST subcellular localization in different cells with two different antibodies. Immunocytochemistry (ICC) assays were performed with two anti-REST sc-25398 (Santa Cruz) and ab21635 (Abcam), which are, respectively, against N and C terminus of REST, for C6, RN46A, and COS7 cells. For each cell line, two wells of cells under the same experimental conditions were stained with sc-25398 and ab21635, respectively. Briefly, cells cultured on poly-D-lysine-coated coverslips were fixed with 4% paraformaldehyde, permeabilized with 0.3% Triton X-100, and incubated with sc-25398 (1:100) or ab21635 (1:200), followed by incubation with a goat anti-rabbit secondary antibody conjugated with Alexa Fluor 568 (1:500, Invitrogen). Nuclei were stained with Hoechst-33342 (Thermo Scientific), and cells were mounted on glass slides. Confocal microscopy was performed using a Leica TCS SP5 Spectral Confocal Microscope. For each cell line, all experimental conditions were kept the same for the two antibodies. Regardless of the cell-types, ICC with sc-25398 yielded predominant localization of REST in nucleus, whereas ICC with ab21635 indicated predominant colocalization of REST with microtubule (or cytoskeleton), suggesting that REST isoforms with different subcellular localization might be differentially recognized by different antibodies.

**Figure 3.**
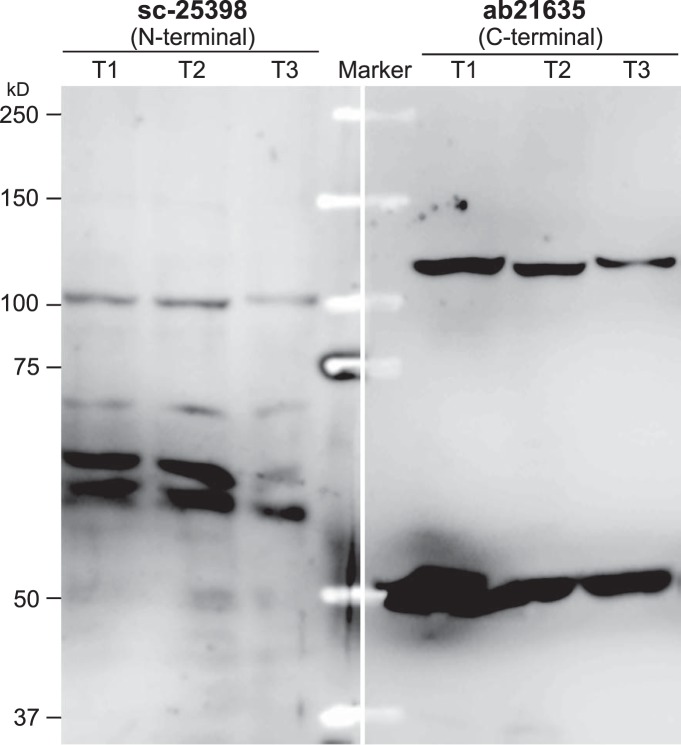
WB of REST expression in HEK-293T cells with two different antibodies. Two aliquots (25 µg for each) of three different HEK293T protein samples (T1, T2, and T3), which were isolated simultaneously with RNA and DNA by TRIzol reagent (Invitrogen), along with a Kaleidoscope marker (Bio-Rad) in between, were loaded on a 7.5% PAGE-SDS gel, followed by electrophoresis and electrotranslocation onto an Immun-Blot PVDF membrane (Bio-Rad), which was then cut into two halves for incubation with sc-25398 (1:250) and ab21635 (1:500), respectively, and subsequent incubation with a goat anti-rabbit IgG (Sigma-Aldrich, 1:2500). IR signals were detected using the VisiGlo Select HRP Chemiluminescent Substrate kit (Amresco) with an ECL-based LAS-3000 image system (Fujifilm). Note that the two antibodies yielded totally different profiles of IR bands.

In their paper describing altered nuclear REST in aging and AD brain, Lu et al. claimed that REST4 mRNA (N_3c_) level in brain tissues comprised only 0.1–0.5% of *REST* mRNA (Lu et al., 2014), while a number of neuronal splice variants produced by ΔE_2_, ΔE_3_, and ΔE_4_ (or inclusion of E_5_; Chen and Miller, 2013), of which ΔE_3_ eliminates a motif critical for nuclear targeting (Shimojo et al., 2001; Shimojo, 2006) and therefore affects nuclear REST (Chen et al., 2017) were not mentioned. It can be simply inferred that if only the full-length REST mRNA exists, all segments of it should share the same level of expression; however, in accordance with the above-mentioned notion of inconsistent results yielded by different primers, qRT-PCR data in Lu et al., indicated that four primer sets (P1–P4) targeting different exons of *REST* yielded strikingly different changes in *REST* mRNA expression. Notably, patterns of this primer-dependent result varied across the aged groups. For instance, P2 assay showed the highest and lowest fold change for the 95-year and >95-year group, respectively, while some assays for aging groups (e.g., P1/P4 for 71 year, P1/P3/P4 for 95 year, and P2 for >95 year) showed mRNA expression levels similar to the 25-year group, suggesting that systematic error made minor contribution to this primer-related discrepancy, which however can be explained by individual variation in alternative *REST* splicing described in our previous study (Chen and Miller, 2013). So, qRT-PCR data presented by Lu et al. actually provided strong evidence for alternative *REST* splicing, which unfortunately was not interpreted in the paper. In addition, unlike Northern blotting, which gives size information for observed mRNAs, qRT-PCR measures abundance of a specific amplicon (i.e., a segment of mRNA), which can be shared by multiple mRNA variants, such that qRT-PCR data may represent expression of multiple splice variants yielding the same amplicon but not merely the full-length *REST* mRNA. Hence, without evidence of Northern blotting, it is difficult to interpret the full-length *REST* mRNA expression level with the primer-dependent qRT-PCR data in Lu et al. Also, given that most of the previously reported mRNA variants were not tested and that the four qRT-PCR primer sets yielded different results, it is unknown how the total mRNA level and the percentage of REST4 mRNA in brain tissues were calculated. Meanwhile, Lu et al. performed a series of experiments (e.g., RNAi, ChIP-seq, and oxidative stress) using the SH-SY5Y cell line, which reportedly expresses abundant REST4 mRNA (N_3c_) and protein (Palm et al., 1999; Yu et al., 2009; Chen and Miller, 2013); however, REST4 expression in SH-SY5Y was not mentioned in the paper.

At the protein level, Lu et al. assayed REST protein expression and subcellular distribution by immunoblotting/immunostaining with a total of six different antibodies, of which two (07-579 and ab52850) and three (ab28018, sc-15118, and IHC-00141) were used for WB and immunohistochemistry (IHC), respectively. As mentioned above, due to the existence of multiple REST protein isoforms, different antibodies may yield different WB/IHC results, while WB with a specific antibody may yield multiple IR bands which represent different REST isoforms sharing the same epitope. So, comparison of the WB/IHC results between different antibodies may hint about the existence of multiple REST protein isoforms; however, no such comparison was shown in Lu et al., while all the presented blots (even for the SH-SY5Y cells with REST4 expression) were maximally cropped with only the band of interest (presumably represents the full-length REST) available, making it impossible to evaluate the potential existence of multiple REST isoforms. Although Lu et al. performed immunostaining to test specificity of one IHC antibody (IHC-00141), the existence of multiple REST isoforms cannot be excluded, because isoforms sharing the same epitope can all bind to the same antibody and this binding can be eliminated by the same blocking peptide.

Notably, it was not disclosed in Lu et al. how the three IHC antibodies were assigned to samples of different groups, giving rise to the concern that nuclear REST differences between the experimental groups might be artificially generated by biased usage of the antibodies for different samples. For example, comparison of nuclear REST between young (*n* = 11), aged (*n* = 77), AD (*n* = 72), and MCI (*n* = 11) groups (Fig. 1*E*, imaging in Lu et al.) was presumably based on staining of the samples with three different antibodies but not a single antibody, otherwise the remaining two antibodies must have been respectively used for another two sets of samples or occasions of experiments, which however were not mentioned in the paper. So, without consideration of differences between the antibodies and disclosure of the antibody usage, the employment of multiple antibodies for IHC did not strengthen findings of Lu et al., but instead introduced an extra confounding variable which made the findings even more questionable.

In response to our doubt about the antibody usage, *Nature* published an addendum on November 16, 2016 (Lu et al., 2016). Specifically, as shown in Table 1, several occasions of IHC experiments, which had not been previously mentioned, were added to the article, making that each antibody was seemingly used on an independent occasion of IHC experiment and that the existence of the above-mentioned confounding effect by misuse of the antibodies was therefore excluded. However, this addendum also raised some concerns. For example, based on the addendum, it can be inferred that all the presented IHC data were obtained using the antibody IHC-00141 but had nothing to do with the other two antibodies (ab28018 and sc-15118) now claimed to have been employed for additional IHC experiments and yielded similar results (“data not shown”); however, all the three antibodies were referred to when the IHC-00141-related results in Figure 1*D*,*E* and Extended Data Figure 1 were mentioned in the paper. Also, if the antibodies ab28018 and sc-15118 yielded similar results as claimed in the addendum, there is no doubt that it would greatly strengthen the data obtained by IHC-00141 and make the findings more convincing; however, it is strange that results of ab28018 and sc-15118 had not been even mentioned in the original article. In other words, there was zero evidence supporting the existence of the IHC experiments that were later added in the addendum without any notification in the statement and any explanation for their absence in the original version of the paper. In addition, like the case for IHC, two antibodies (07-579 and ab52850) were previously listed for WB without disclosure of their usage for each independent experiment; however, based on the addendum, all the presented WB data were obtained using 07-579, while neither the usage nor the result information was disclosed for ab52850, raising the question why this antibody was listed in the paper.

**Table 1.** A summary of IHC assay mentioned in Lu et al., with and without the addendum

Antibody	Without addendum	With addendum			
	IHC-00141, ab28018, sc-15118	IHC-00141	ab28018	sc-15118	ab202962
Sample (*n*)					
Total	171	171	7 of 171	49 of 171	35 of 171
Young	11	11	?	10	10
Aged	77	77	?	21	14
AD	72	72	?	18	11
MCI	11	11	?	-	-
Result	All presented IHC data	All presented IHC data	Data not shown	Data not shown	Data not shown

Even if a fixed antibody was used for both IHC and WB throughout the study, expression of multiple REST isoforms caused by alternative splicing still may lead to data misinterpretation. For example, REST4, expression of which in SH-SY5Y was ignored by Lu et al., competes with the full-length REST to occupy RE-1 sites, such that it inevitably affects interpretation of REST target genes with the ChIP-seq data. Also, REST isoforms sharing the same epitope can be indiscriminately labeled by a specific antibody, and, in comparison with the full-length REST, truncated isoforms presumably have less complexity of protein folding and three-dimensional structure which potentially masks the epitope and therefore they may be more accessible by the staining antibody. As mentioned above, test of antibody specificity by immunostaining does not help to exclude the existence of multiple REST isoforms sharing the same epitope, whose binding to the antibody can be eliminated by the same blocking peptide. So, the IHC results could not address which specific REST isoform(s) contributed to differences in nuclear REST between the experimental groups; however, with only the full-length REST having been considered, such differences were attributed to the full-length REST in Lu et al. Taken together, Lu et al. neglected previously documented REST isoforms which presumably confound experimental results and lead to data misinterpretation (e.g., qRT-PCR data), while the usage of multiple antibodies for REST protein assay is questionable, making it necessary for a reevaluation of their findings.

## Synthesis

Reviewing Editor: Jörg Schulz, RWTH Aachen University Decisions are customarily a result of the Reviewing Editor and the peer reviewers coming together and discussing their recommendations until a consensus is reached. When revisions are invited, a fact-based synthesis statement explaining their decision and outlining what is needed to prepare a revision will be listed below. The following reviewer(s) agreed to reveal their identity: Judith Stegmueller, Avtar Roopra.

The article by Chen and Miller argues for a more comprehensive analysis of REST splicing - specifically in Lu et al 2014. Chen and Miller provide a detailed summary of the known, validated REST variants as well as some putative variants that may exist. This is an important piece of work as there are some concerns in the field regarding some of the claims in Lu et al. Chen and Miller focus on REST splicing which is appropriate given that the bar graph in figure 1b of Lu et al is critical to the manuscript yet shows inexplicable differences in REST levels depending upon the primer pair used. This coupled with the poor schematic detailing where the primers target REST, is a reasonable basis to query this figure. Chen and Miller also show typical westerns with 2 common REST antibodies. The whole blot is shown and therefore all the bands are visible. The blots show the messy nature of REST blots that we all see but that rarely get published. Similarly, Immunofluorescence using the 2 antibodies shows very different cellular localization, again highlighting the care with which such images should be interpreted. This is especially important given the known shuttling of REST between the nucleus and cytoplasm. My concerns with current manuscript mainly focus on the writing style. The paper is hard to read due to poor grammar throughout. Examples are: ‘Supported by that immunoblotting/-staining with different anti-REST antibodies yield inconsistent results, alternative splicing allows production of various structurally and functionally different REST protein isoforms in response to shifting physiological requirements, providing a reasonable explanation for the diverse, highly context-dependent REST function.’ “it was claimed in the addendum that 7 cases from the 171 cases labeled with IHC-00141 were labeled in separate slides with ab28018 and that ‘similar patterns of REST immunoreactivity were observed with increased nuclear REST in neurons in aged versus young adult cases and reduced nuclear REST in AD’, but the sample size (7 cases comprising at least 3 experimental groups, i.e. n&lt;3 for 1-2 groups) is extremely too small to perform statistics”

## Author Response

Nature Author #1 (Remarks to the Author)

The commentary by Chen and Miller suggests that alternative splice variants in REST may influence our findings. However, these authors have not performed any of the experiments in our paper (1) and do not provide evidence that alternative splice variants of REST contribute to our findings. Even without any new data, we can say with confidence that alternative REST splicing was underappreciated in a number of previous publications including Lu et al. What makes the paper of Lu et al. doubtful is that the authors ignored previously documented REST splice variants and that the employment of multiple anti-REST antibodies in their study is questionable, giving rise to the concern that nuclear REST difference between the experimental groups (young, aged, and AD) might be artificially generated by selective or preferential use of antibodies for different samples. Our concern was actually strengthened but not eliminated by the authors response to our commentary. 1. Chen and Miller note that they have described 45 or more potential alternative splice variants of REST in a previous publication (2) and that we have not considered these variants. However, most of these potential variants have not been shown to be translated into functional proteins. In our paper, we showed that a major established REST splice variant in brain, REST4, comprised &lt;0.5% of REST mRNA in adult human prefrontal cortex. Chen and Miller suggest that we did not exclude expression of a putative new REST exon, “exon 5”, described by these authors. However, this new exon has also not been validated, and its theoretical presence would not change our conclusion that REST protein containing the canonical N- and C-terminal domains is increased in the aging human brain and decreased in AD. Chen and Miller also note that RT-PCR data in Fig. 1b of our paper “support the existence of alternative splicing in the aging brain”. This is misleading as there is no consistent change in the relative levels of PCR products of the different primers among aged brain samples to support an age-related change in REST splicing. The only consistent change is significantly increased PCR product, consistent with our conclusion that REST mRNA is increased in the aged brain. All the new REST exons and splice variants reported in our previous paper were sequence verified and deposited in NCBI GenBank, and primers for PCR assay of specific splice variants were clearly indicated in related Figures. So, I do not understand why the authors say that exon 5 is putative and has also not been validated. As mentioned in our commentary, not all REST protein isoforms predicted by alternative splicing have been validated; however, it is true that multiple REST protein isoforms (REST4, REST1 and RESTC) have been described. Particularly, the existence of RESTC provided strong evidence that E2-lacking mRNA variants (e.g. E1/E3/E4), which are widely expressed in mammalians and resistant to conventional REST knockout strategy (excision of E2), can be translated into functional proteins by using an alternative translation start site. As for the exon 5, it is predictive of REST4 which may confound findings of Lu et al. as mentioned below. Thus, we propose that alternative REST splicing was previously underappreciated, and that REST isoforms might be differentially assayed or manipulated, resulting in conflicting findings on REST. This presumption is well supported by that different anti-REST antibodies yield inconsistent immunoblotting/- staining results, for which we presented data for two antibodies (sc-25398 and ab21635) in our commentary. The RT-PCR data (Fig.1b) in Lu et al. clearly showed that expression of the 4 targeted regions (P1-P4) of REST changed differentially in most aged groups (71, 80, 88, 95 and &gt;95 yr), and some regions (P1 and P4 in 71-yr, P1, P3 and P4 in 95-yr, and P2 in &gt;95-yr) displayed similar levels of expression with that of the 25-yr groups. That is, P1-P4 regions were not expressed at the same level in aged groups. If only the full-length mRNA is expressed without alternative splicing, how can this differential expression be explained? By the way, we never claimed that a consistent expression change should be observed across the aged groups, but instead, we mentioned individual variation in alternative REST splicing in our previous study. 2. Chen and Miller claim that the REST4 splice variant might contribute to our findings in SH-SY5Y cells. This variant accounts for ~10-15% of the REST transcripts in SY5Y cells. It is important to note that an integral part of the experiments in SY5Y cells was overexpression of REST by transduction of REST cDNA. REST cDNA cannot generate the REST4 splice variant. The mRNA and protein expression of REST4 in SH-SY5Y cells, which was reported by 3 different groups, was totally ignored in Lu et al. Now the authors claim that REST4 accounts for ~10-15% of REST transcripts in SH-SY5Y cells. First, it is questionable how percentage of a specific isoform was calculated without considering other previously documented splice variants (i.e. E1/E3/E4). Second, the authors mentioned above that most of the REST mRNA variants identified in our previous study have not been shown to be translated into functional proteins, which means protein expression is more important than mRNA evidence; however, it is strange that without exception, the blot area informative of REST4 protein (predicted as 37kD but observed as 53kD) was excluded from all the immunoblots presented in Lu et al., implying that the authors had no intention to clarify whether or not REST4 protein is expressed in the tissue and cell samples, although REST4 expression in SH-SY5Y had been established. The authors claim that REST4 expression in SH-SY5Y will not affect their findings because REST overexpression was examined as a positive control. First, REST overexpression was not examined in some experiments (e.g. autophagy and FOXO1 expression). Second, in terms of REST knockdown, both REST4 and the full-length REST are targeted by the two shRNAs, how can the shRNA-induced effects be attributed to the full-length REST only? Third, as mentioned below, REST4 may be stained as same as the full-length REST. Moreover, alternative splicing is epigenetically regulated while REST itself is an epigenetic regulator, what if REST4 expression changes in response to REST overexpression or knockdown? So, as mentioned in our commentary, REST4 might be a major confounding factor in Lu et al. and its expression should not be ignored. 3. Chen and Miller suggest that the multiple REST antibodies we employed were “indiscriminate”. These antibodies were chosen to span the N- and C-terminal domains of the REST protein, and were screened by multiple antibody specificity controls, as described in our paper. In contrast, the immunocytochemical labeling presented in the Chen and Miller commentary (Fig. 2) is lacking in any specificity controls, used antibodies that were not used in our paper, and was performed in cell types not used in our paper. The question of antibody specificity is especially pertinent to their labeling with REST antibody ab21635, which they claim shows REST “colocalization with microtubules” (Fig. 2). To our knowledge, this has never been reported for REST. Chen and Miller cite 2 publications as evidence for this, which is misleading as the cited papers do not show this labeling pattern. Instead of clarifying how the multiple REST antibodies were assigned to each staining, which usually should be clearly indicated in the figure or legend, the authors insist that the antibodies employed in their study can exclusively recognize the full-length REST because they tested specificity of the antibodies. However, specificity data shown in Lu et al. was insufficient to exclude the existence of REST isoforms. As we all know, immunoblotting/-staining is based on binding of the antibody to its specific epitope, which however can be shared by multiple protein isoforms. For example, REST4, REST1 and the full-length REST share the same epitope (100-150AA) for sc-15118, such that: 1) all of the 3 isoforms can be indistinguishably stained by sc- 15118; 2) following electrophoresis separation and blotting with sc-15118, they can be observed as immunoreactive bands of different sizes; and 3) peptide(s) blocking the binding of sc-15118 to its epitope will work for all of the 3 isoforms. That is, REST isoforms sharing the same epitope can only be discriminated by blotting, but not by staining. Lu et al. tested specificity of immunostaining for 2 antibodies (IHC-00141 and 07-579?), but did not perform immunoblotting to exclude the existence of multiple REST isoforms which contain epitopes of the IHC antibodies. Thus, the existence of multiple REST protein isoforms, which are indistinguishable by immunostaining, was actually not excluded in Lu et al. Due to the existence of multiple REST isoforms, different antibodies may yield different staining patterns, like the data shown in our commentary. In our opinion, to avoid data misinterpretation, at least one antibody should be consistently used for samples of different groups while additional antibodies may be employed for data validation, otherwise the use of multiple antibodies will give rise to the concern that differences between the experimental groups might be artificially generated by selective or preferential use of the antibodies. It is notable that Lu et al. employed 3 antibodies (sc-15118, IHC-00141 and ab28018) for IHC but did not compare staining patterns between the antibodies, implying that the employment of multiple antibodies was not used for data validation. As such, what is the rationale for using multiple antibodies in their study? Lu et al. did not clearly indicate how the 3 IHC antibodies were assigned to each staining, making it possible that the antibodies were preferentially used for samples of different groups. Interpretation of immunoblotting/-staining results should consider not only the full-length REST, but also potential REST isoforms (e.g. REST4, REST1 and RESTC). Notably, in accordance with loss of conservation for internal sequences of exon 4, we previously showed that partial or complete skipping of exon 4 is very common, suggesting that the full-length REST might not be predominantly expressed. Although it is unknown whether non-specific binding contributes to co-localization of REST with microtubule stained by ab21635, it has been documented that REST associates with other factors (e.g. Huntingtin and dynactin p150Glued, of which the latter binds to microtubule) to form a cytoskeletal complex, which prevents REST from being translocated into nucleus. As an antibody against C-terminus of REST, ab21635 will recognize not only the full-length REST, but also truncated C-terminal REST isoforms (e.g. RESTC). Lacking zinc finger-5 which is reportedly important for nuclear targeting, the Cterminal REST isoforms may be predominantly localized in cytoplasm but still be capable of entering the nucleus through the putative NLS. In addition, our unpublished data show that staining with ab21635 responds to manipulation of alternative REST splicing. Thus, we would rather not exclude the possibility that a proportion of REST (full-length or truncated) co-localize with microtubules. 4. Chen and Miller suggest that our results on REST-KO mice could be confounded by the expression of a Cterminal REST isoform generated by skipping exon 2 with residual biological activity. We used the same REST gene targeting strategy utilized in the original REST-KO report and subsequently used by other labs (3,4). Furthermore, our paper showed that the vulnerability phenotype of neurons derived from Nestin- Cre:REST-KO mice was rescued by expression of wild-type REST, confirming REST loss of function. If there were some residual REST function in the REST-KO, it would suggest that the phenotype we observed is an underestimation of the true neuroprotective activity of REST. As such, it would not change any of the conclusions. It is still largely unknown about the diverse, context-dependent function of REST with numerous contradictions, so its not surprising that new findings in this field may greatly update our knowledge of REST. The existence of multiple REST mRNA and protein isoforms, which are yielded by alternative splicing, strongly suggests that REST isoforms might have been differentially assayed or manipulated in previous studies; however, in most cases, only the full-length REST was considered for data interpretation. With the remaining expression of RESTC isoform in conventional REST-KO mice having been recently described, we have no doubt that previous findings obtained from such kind of mice should be re-examined. Again, what makes the paper of Lu et al. doubtful is that the authors ignored previously documented REST splice variants and that the employment of multiple anti-REST antibodies in their study is questionable, giving rise to the concern that nuclear REST difference between the experimental groups might be artificially generated by selective or preferential use of antibodies.
